# Efficacy and safety of apatinib versus sorafenib/placebo in first-line treatment for intermediate and advanced primary liver cancer: A systematic review and meta-analysis

**DOI:** 10.3389/fphar.2023.1101063

**Published:** 2023-04-21

**Authors:** Dan Peng, Yongqing Cai, Geng Chen, Min Hou, Xiaofeng Luo, Zhuoma Dongzhi, Hongjun Xie, Yao Liu

**Affiliations:** ^1^ Department of Pharmacy, Army Medical Center, Chongqing, China; ^2^ Department of Hepatology, Army Medical Center, Chongqing, China; ^3^ Medical College of Tibet University, Lhasa, China

**Keywords:** apatinib, sorafenib, primary liver cancer, meta-analysis, systematic review

## Abstract

**Background:** Apatinib is a novel tyrosine kinase inhibitor used in the treatment of advanced hepatocellular carcinoma (HCC). For decades, sorafenib has been a classic first-line treatment option for patients with HCC. This meta-analysis aimed to assess the efficacy and safety of apatinib versus sorafenib/placebo as first-line treatment for intermediate and advanced primary liver cancer (PLC).

**Methods:** A literature search was performed *via* PubMed, Web of Science, CENTRAL, Embase, CNKI, VIP, and CBM. Data extraction from databases of other languages is not restricted. The Cochrane risk of bias tool, modified Jadad scale, Newcastle–Ottawa scale (NOS), and non-randomized studies of interventions (ROBINS-I) tool were employed to evaluate methodological qualities in original studies. Influence analysis was applied to assess the reliability of pooled results. Publication bias was evaluated using the funnel plot with Begg’s test and Egger’s test.

**Results:** Seven studies were included in the systematic review and meta-analysis. Four randomized controlled trials (RCTs) and one clinical controlled trial (CCT) were used for comparing apatinib with placebo, and two retrospective clinical studies (RCSs) were used for comparing apatinib with sorafenib. Apatinib led to higher overall effects in objective response rate (ORR), disease control rate (DCR), and mean survival time (MST) over placebo (RR = 2.03, 95% CI = 1.46–2.81, *p* < 0.0001, I^2^ = 0%; RR = 1.17, 95% CI = 1.04–1.33, *p* = 0.009, I^2^ = 45.8%; SMD = 2.63; 95% CI = 1.47–3.78, *p* < 0.0001, I^2^ = 92.7%, respectively). Compared to sorafenib, apatinib showed no superiority in ORR and DCR but was inferior in the 6-month and 1-year survival rate (RR = 1.99, 95% CI = 0.85–4.65, *p* = 0.111, I^2^ = 68.3%; RR = 1.04, 95% CI = 0.73–1.47, *p* = 0.840, I^2^ = 0.0%; RR = 0.63, 95% CI = 0.42–0.97, *p* = 0.036, I^2^ = 0.0%; RR = 0.47, 95% CI = 0.29–0.79, *p* < 0.0001, I^2^ = 0.0%, respectively). Apatinib had similar adverse effects over placebo but possessed a greater incidence rate of proteinuria and hypertension over sorafenib.

**Conclusion:** In the first-line setting, apatinib might be an alternative treatment approach for patients with intermediate and advanced PLC. Sorafenib alone showed a better survival rate within 1 year and a lower incidence rate in hypertension and proteinuria than apatinib monotherapy.

## Introduction

Primary liver cancer (PLC) remains one of the five most common malignant neoplasms worldwide and is ranked as the second cause of cancer mortality in China (Bray et al., 2018; Petrick and Mcglynn., 2019). Hepatocellular carcinoma (HCC) comprised over 75% of cases with PLC (Dasgupta et al., 2020). Patients are diagnosed with PLC commonly in the intermediate and advanced stages. To date, tyrosine kinase inhibitors (TKIs), anti-angiogenic agents, and immune checkpoint inhibitors (ICIs) are the chief systematic treatment for HCC.

TKIs such as sorafenib ushered in the era of systemic therapy for HCC. At present, TKIs are still considered the backbone of HCC treatment, especially in patients with autoimmune disorders and transplantation who are not appropriate for immunotherapy (da Fonseca et al., 2020). Sorafenib as the classical TKI was approved by the Food and Drug Administration (FDA) in 2007 and recommended as first-line targeted agent for patients with HCC (Heimbach et al., 2018; Vogel and Martinelli., 2021). Sorafenib showed a moderate survival benefit with a mild and manageable toxicity profile (Llovet et al., 2008; Cheng et al., 2009). Although sorafenib might not currently be the optimal treatment approach, it was considered the most common first-line therapeutic agent for patients with advanced HCC. So far, sorafenib has been approved for marketing in more than 100 countries worldwide and has benefited more than 1 million people.

However, only 30% of patients with HCC could benefit from TKIs, which are prone to resistance (Chidambaranathan-Reghupaty et al., 2021). The prognosis of therapies with TKIs was yet short of expectation, and patients had a five-year survival rate of merely 12.5% (Bruix et al., 2016). Apatinib, a new selective inhibitor of VEGFR2 tyrosine kinase and an anti-angiogenic medication, showed satisfactory efficiency in unresectable HCC or advanced liver carcinosarcoma (Liu et al., 2018; Zhao et al., 2019). In October 2020, apatinib as the second-line therapy for patients with HCC was approved by the National Medical Products Administration (NMPA). For the rest of the world except China, apatinib was relatively little known. Clinical trials on apatinib versus placebo/sorafenib were conducted for patients with PLC in a first-line setting; however, these results were not straightforward in primary and secondary outcomes (Zhou, 2019; Xu et al., 2018). In 2020, a network meta-analysis evaluated the comparative effectiveness of different systemic treatments including linifanib, sunitinib, brivanib, lenvatinib, and atezolizumab–bevacizumab versus sorafenib in patients with advanced HCC in a first-line setting (Sonbol et al., 2020). The current systematic review aimed to assess the efficacy and safety of a novel TKI (apatinib) versus a conventional TKI (sorafenib). Beyond that, compared to sorafenib or other systemic therapies, apatinib showed an added advantage of cost-efficiency (Scott, 2018). It would be a feasible option for many patients with PLC who cannot afford existing systemic therapy, especially in many developing countries.

Thus, we performed a large-scale analysis of clinical studies for comparing the therapeutic effects and safety of apatinib versus placebo or sorafenib in first-line treatment of patients with intermediate and advanced PLC. Systematic evaluation for the merits and demerits of apatinib, including short-term efficacy, long-term survival benefits, and adverse reaction, would be helpful for clinicians to make a reasonable treatment option.

## Methods

### Literature search

A comprehensive literature search in seven databases, including PubMed, Web of Science, Cochrane Central Register of Controlled Trials (CENTRAL), EMBASE, China National Knowledge Infrastructure (CNKI), Chinese Science and Technology Journals database (VIP), and Chinese Biological Medicine database (CBM), was performed from the first available date to 08 September 2022, using the following search terms: “apatinib” or “sorafenib,” “hepatic carcinoma” or “hepatocellular carcinoma” or “liver cancer” or “primary liver cancer.” The terms were revised with the requirements of other databases. Any restrictions of different languages were not applied in this meta-analysis. Furthermore, a manual search of other registers was performed to filter out potential eligible studies. This systematic review conformed to the Preferred Reporting Items for Systematic Reviews and Meta-Analyses (PRISMA) guidelines.

### Criteria for inclusion and exclusion

The inclusion criteria included clinical studies with (I) the number of subjects in each group >10; (II) all subjects belonging to intermediate and advanced PLC; III) subjects conformed to at least one of the following criteria: Barcelona Clinic Liver Cancer (BCLC) stage B or C, China Liver Cancer (CNLC) stage III or Ⅳ, or the intermediate–advanced stage by other major staging criteria for PLC worldwide; (Ⅳ) adequate baseline data of subjects; (Ⅴ) the Karnofsky (KPS) scores of subjects >60; (Ⅵ) apatinib alone as the intervention group; (Ⅶ) placebo or sorafenib as the comparison group; (Ⅷ) apatinib or sorafenib in the first-line setting; and (Ⅸ) outcome measures including at least two of the following items: the efficacy indicators of solid tumors including disease control rate (DCR) and objective response rate (ORR), lifetime for subjects containing mean survival time (MST) and survival rate (SR), and adverse effects.

The exclusion criteria were as follows: (I) subjects who had received chemotherapy, radiotherapy, or biotherapy within 1 month prior to the clinical trial; (II) subjects with severe heart and mental illness; (III) clinical studies in the refractory settings; (Ⅳ) studies about combination treatment; (Ⅴ) any publication with incomplete data; (Ⅵ) irrelevant topics, review articles, and duplicate literature; and (Ⅶ) single-arm and case–control trials.

### Data extraction

The data of each study were independently extracted by two review authors (DP and YC) using a normative form. Partial disputes were resolved by discussing with a third review author (GC). Meantime, the third reviewer (GC) double-checked data extraction and compared the results. A corresponding author (YL) was included to settle the residual and difficult disputes. For the included studies, we extracted the characteristics of studies, short clinical response, MST, 6-month or 1-year SR, and adverse effects. The baseline characteristics of studies included first author, study design, group, starting dose, sample size, duration of treatment, follow-up period, the number of male and female patients, age, tumor size, baseline AFP levels, and outcome indexes. The response evaluation criteria in solid tumors (RECIST) was applied to evaluate the short-term tumor response, including ORR and DCR.

### Outcomes

The primary outcomes were MST and 6-month or 1-year SR. The secondary outcomes included ORR and DCR. Meanwhile, safety was evaluated with the treatment-related adverse events.

### Quality evaluation and bias risk assessment

The Cochrane “risk of bias” assessment tool was used independently by two reviewers (DP and GC) in randomized controlled trials (RCTs). The results were categorized into low, unclear, or high risk of bias. The bias items for each study included random sequence generation (selection bias), allocation concealment (selection bias), blinding of participants and personnel (performance bias), blinding of outcome assessment (detection bias), incomplete outcome data (attrition bias), selective reporting (reporting bias), and other bias.

The revised Jadad scale was applied independently by two reviewers (DP and GC) to assess the methodological qualities of RCTs. The Jadad scale has a maximum score of 7. The study with less than 4 scores was considered to be of low quality and that with more than 4 scores indicated high quality. The Newcastle–Ottawa scale (NOS) was applied to evaluate the methodological qualities of controlled clinical trials (CCTs) and retrospective clinical studies (RCSs) from the selection of research objects, comparability between groups, and measurement of exposure factors. All disagreements between the two reviewers were settled through consulting with a third reviewer (YL).

The non-randomized studies of interventions (ROBINS-I) tool was applied to evaluate the risk of bias in non-randomized studies in seven domains: bias due to confounding, bias in selection of participants, bias in classification of interventions, bias due to departures from intended interventions, bias from missing data, bias in measurement of outcomes, and bias in selection of the reported result.

### Statistical analysis

The meta-analyses were performed using Stata/MP (version 14.0; StataCorp LP, College Station, TX) software. Dichotomous data were presented as risk ratio (RR) with 95% confidence interval (CI). Continuous data were expressed as standardized mean difference (SMD) with 95% CI. Statistical heterogeneity was presented with chi-squared and *I* ^*2*^ values. I^2^ > 50% indicated the evident heterogeneity of studies included, and the random-effect model was used to analyze the overall outcomes. If heterogeneity was not significant, the fixed-effect model was applied. The leave-one-out method for influence analysis was applied to assess the reliability of results in this meta-analysis. Publication bias was evaluated using the funnel plot with Begg’s test and Egger’s test. *p*-value <0.05 was considered to be statistically significant.

## Results

### Baseline characteristics of the included studies

**FIGURE 1:**
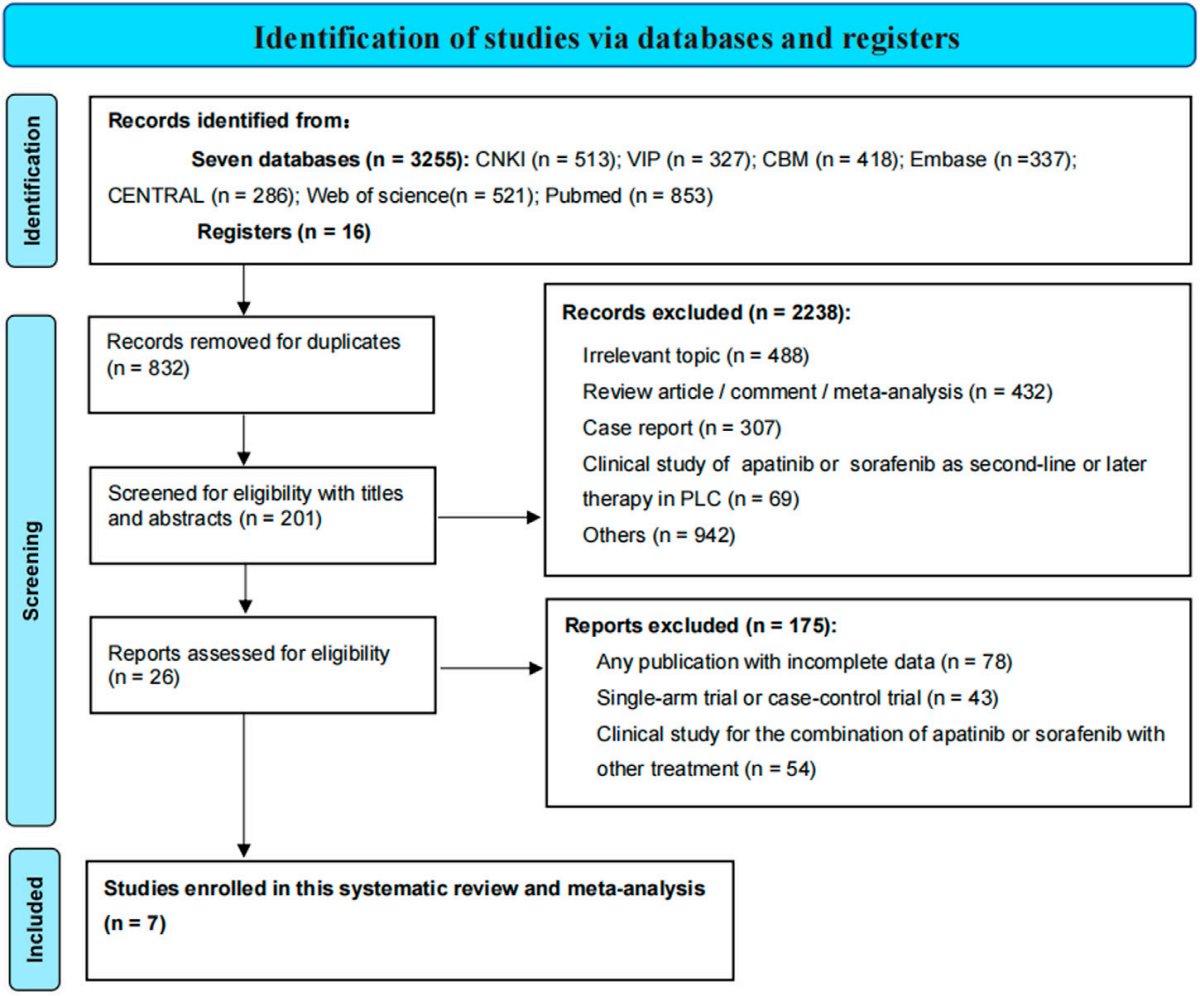
PRISMA flowchart for selection of relevant studies in this meta-analysis.

We screened a total of 3,271 publications through database searching. Seven studies in the first-line setting were lastly included in our meta-analyses (Liao et al., 2017; Wang et al., 2018; Xu et al., 2018; Zheng et al., 2018; Wei et al., 2019; Zhou, 2019; He et al., 2020). Four RCTs and one CCT were used for comparing apatinib with placebo (Liao et al., 2017; Xu et al., 2018; Zheng and Lin, 2018; Wei et al., 2019; Zhou, 2019), and two RCSs were used for comparing apatinib with sorafenib (Wang et al., 2018; He et al., 2020). Detailed information on the selected studies is presented in Figure 1. This meta-analysis encompassed data from 510 patients, of which 300 were included in the studies of apatinib versus placebo and 210 in the studies of apatinib versus sorafenib. These patients had intermediate and advanced PLC and were all from Chinese mainland. The average age of the subjects was more than 50 years. In clinical trials comparing apatinib with placebo, the dose of oral apatinib was 750 mg and 850 mg per day. Placebo was similar in shape and color to those of oral apatinib. In studies of apatinib versus sorafenib treatment, the dose of oral sorafenib was 800 mg per day and the dose of oral apatinib was 500 mg and 750 mg per day. Detailed information about each trial is summarized in Table 1.

**TABLE 1:** Baseline characteristics of patients in the included studies.

Author (year)	Study design	Types of tumor	Group	Starting dose	Sample size	Duration, months	Follow-up, months	Age, years	Tumor size, cm	AFP, μg/L	Outcome index
Wei and ya (2019)	RCT	PLC	Apatinib	425 mg, bid	25	3	NA	58.5 (7.0)	6.1 (1.2)	24.8 (4.7)	DCR, ORR, MST, AEs
			Placebo		25			58.2 (7.1)	6.2 (1.3)	24.6 (4.6)	
Zheng and Lin (2018)	RCT	PLC	Apatinib	750 mg, qd	30	3	3	61.5 (8.4)	NA	28.5 (11.7)	DCR, ORR, MST, AEs
			Placebo		30			60.9 (8.9)		26.7 (12.1)	
Liao et al. (2017)	RCT	HCC	Apatinib	850 mg, qd	30	NA	NA	58.5 (7.2)	6.1 (1.1)	25.2 (5.0)	DCR, ORR, MST, AEs
			Placebo		30			58.2 (6.6)	6.3 (1.3)	24.0 (4.7)	
Zhou (2019)	RCT	PLC	Apatinib	750 mg, qd	29	2	6	61.1 (6.3) 59.1 (4.7)	NA	NA	DCR, ORR, MST, AEs
			Placebo		29						
Xu et al. (2018)	CCT	PLC	Apatinib	850 mg, qd	36	NA	12	58.2 (6.6)	6.3 (1.3)	24.1 (4.8)	DCR, ORR, MST, AEs
			Placebo		36			57.9 (6.3)	6.2 (1.1)	24.3 (4.8)	
Wang et al. (2018)	RCS	HCC	Apatinib	750 mg, qd 400 mg, bid	34	NA	4	53.32 (12.19)	NA	56.0 (12.4)	DCR, ORR, SR, AEs
			Sorafenib		38			50.63 (10.22)		35.0 (17.4)	
He et al. (2020)	RCS	HCC	Apatinib	500 mg, qd	26	NA	13.2 (5.7–20.7)^a^	48.1 (42.3–53.0)^a^	6.1 (2.4–9.7)^a^	39.8 (6.8–765.6)^a^	DCR, ORR, SR, AEs
			Sorafenib	400 mg, bid	46			52.6 (44.8–61.9)^a^	5.8 (2.6–9.0)^a^	39.8 (15.0–1069.0)^a^	

Abbreviations: RCT, randomized controlled trial; CCT, controlled clinical trial; RCS, retrospective clinical study; PHC, primary liver cancer; HCC, hepatocellular carcinoma; qd, quaque die; bid, bis in die; ORR, objective response rate; DCR, disease control rate; MST, mean survival time; SR, survival rate; AFP, alpha fetoprotein; AEs, adverse events; ^a^, interquartile range values between brackets; NA, not available.

### Quality assessment

**FIGURE 2:**
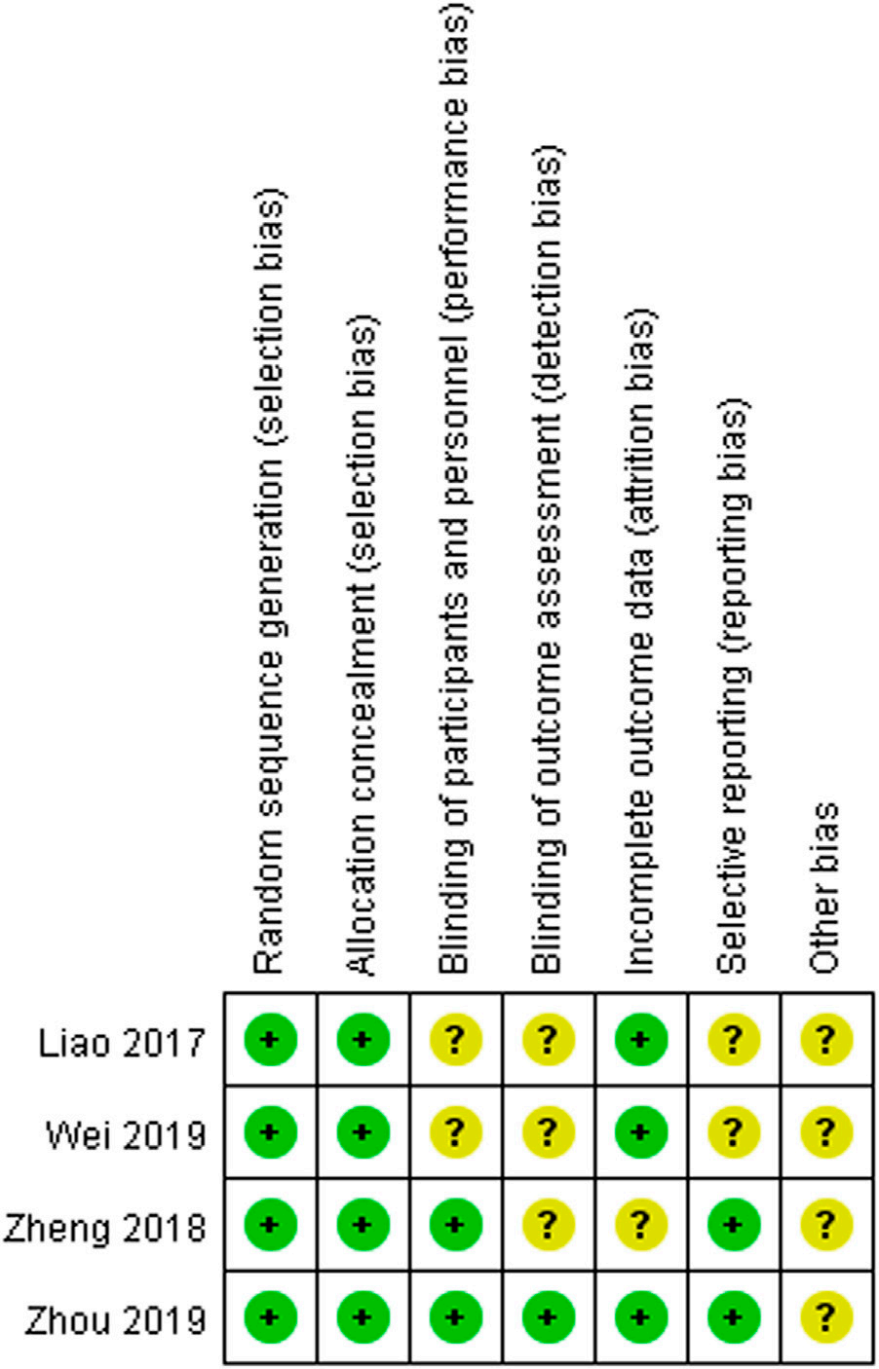
Bias assessment of randomized controlled trials.

The risk of bias assessment of reference was conducted using the Review Manager (version 5.4) software. The bias assessment of four RCTs is summarized in Figure 2. From the results, we found that four studies had a low risk of bias in random sequence generation and allocation concealment. The performance and reporting bias of two RCTs showed a low risk. The attrition bias in three RCTs was considered to be low risk. The detection bias of only one RCT remained unclear. The other sources of bias were unclear across all the original RCTs.

All four RCTs described the blinding and allocation concealment methods. The randomization method was described in three RCTs. Only two studies reported the deviations and drop-out rates of subjects in detail. The basic characteristics and quality scores of RCTs are shown in Table 2.

**TABLE 2:** Quality assessments of randomized controlled trials.

Author (year)	Randomization	Concealment of allocation	Double blinding	Withdrawals and dropouts	Modified Jadad score
Wei and Ya (2019)	The randomization was described, and it was appropriate	The study was described as using the allocation concealment method	The study was described as double-blind	Follow-up was not described	4
Zheng and Lin (2018)	The study was described as randomized	The study was described as using the allocation concealment method	The study was described as double-blind	A description of withdrawals and dropouts	4
Liao et al. (2017)	The randomization was described, and it was appropriate	The study was described as using the allocation concealment method	The study was described as double-blind	Follow-up was not described	4
Zhou (2019)	The randomization was described, and it was appropriate	The study was described as using the allocation concealment method	The study was described as double-blind	A description of withdrawals and dropouts	5

The remaining three studies were one CCT and two RCSs, which were assessed using NOS and the ROBINS-I tool, respectively. In quality assessment with NOS, all of these studies were high quality (4–5 points). Detailed information on the quality assessments of the CCT and RCSs is summarized in Table 3. In quality evaluation using the ROBINS-I tool, three studies showed an overall risk of bias identified as “moderate”. The results of each study are reported in Table 4.

**TABLE 3:** Quality assessments of the controlled clinical trial and retrospective clinical studies.

Author (year)	Selection	Comparability	Exposure	NOS score
Xu et al. (2018)	★★★	★	★★	6
Wang et al. (2018)	★★★	★★	★	6
He et al. (2020)	★★★	★★	★★	7

Abbreviation: NOS, Newcastle–Ottawa scale.

**TABLE 4:** Risk of bias of each domain and overall risk of bias in non-randomized studies (ROBINS-I).

Author (year)	Confounding	Selection	Intervention classification	Measurement of intervention	Missing data	Measurement of outcome	Reported result	Overall
Xu et al. (2018)	——	——	——	——	?	——	+	Moderate
Wang et al. (2018)	+	——	——	+	?	+	+	Moderate
He et al. (2020)	+	——	+	+	+	+	+	Moderate

+: Low, comparable to a well-performed randomized trial.

——: Moderate, sound for a non-randomized study, but not comparable to a rigorous randomized trial.

?: Insufficient information provided to determine risk of bias.

## ORR and DCR

**FIGURE 3:**
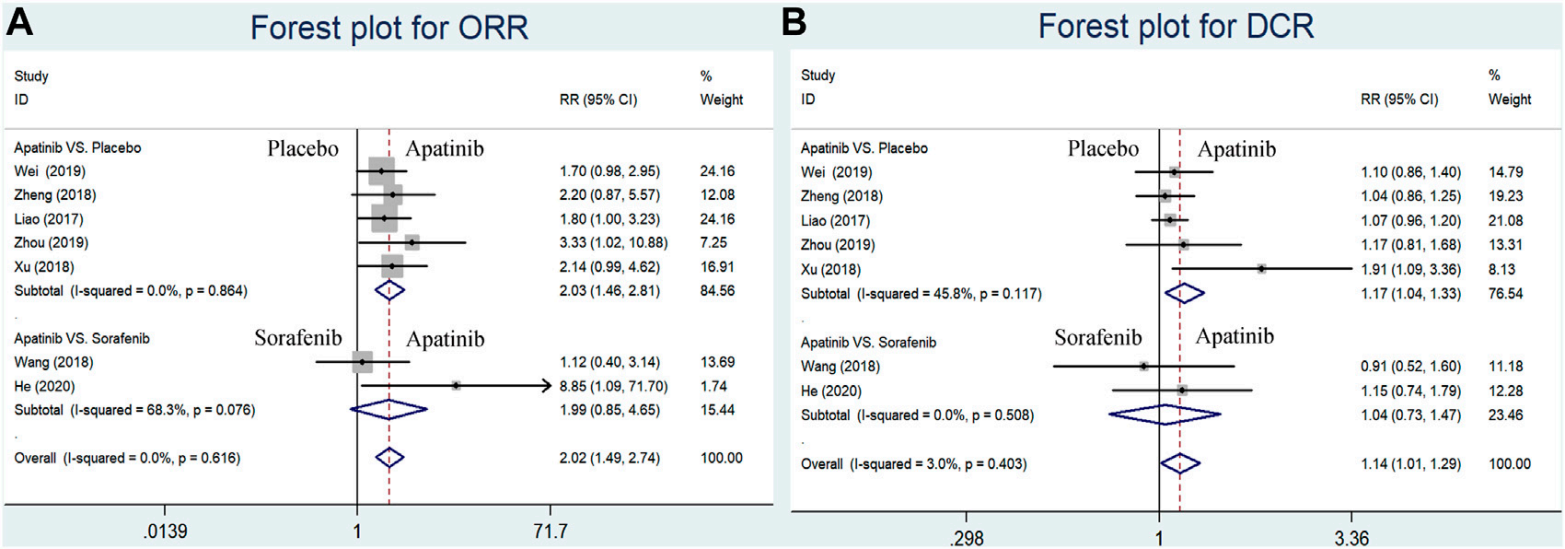
Forest plots for ORR and DCR for included studies comparing apatinib with either placebo or sorafenib in the first-line setting. **(A)** Meta-analysis for the ORR; **(B)** meta-analysis for the DCR. RR indicates the ratio of the incidence of each outcome; 95% confidence interval is presented as error bars.

The short-term efficacy indicators including ORR and DCR were reported in seven studies (four RCTs and one CCT comparing apatinib with placebo and two RCSs comparing apatinib with sorafenib). Briefly, ORR and DCR were significantly higher in the apatinib group than in the placebo group (RR = 2.03, 95% CI = 1.46–2.81, *p* < 0.0001, I^2^ = 0%; RR = 1.17, 95% CI = 1.04–1.33, *p* = 0.009, I^2^ = 45.8%, respectively) (Figures 3A,B). Apatinib had no evident difference in ORR or DCR compared to sorafenib (RR = 1.99, 95% CI = 0.85–4.65, *p* = 0.111, I^2^ = 68.3%; RR = 1.04, 95% CI = 0.73–1.47, *p* = 0.840, I^2^ = 0.0%, respectively) (Figures 3A, B).

### MST

**FIGURE 4:**
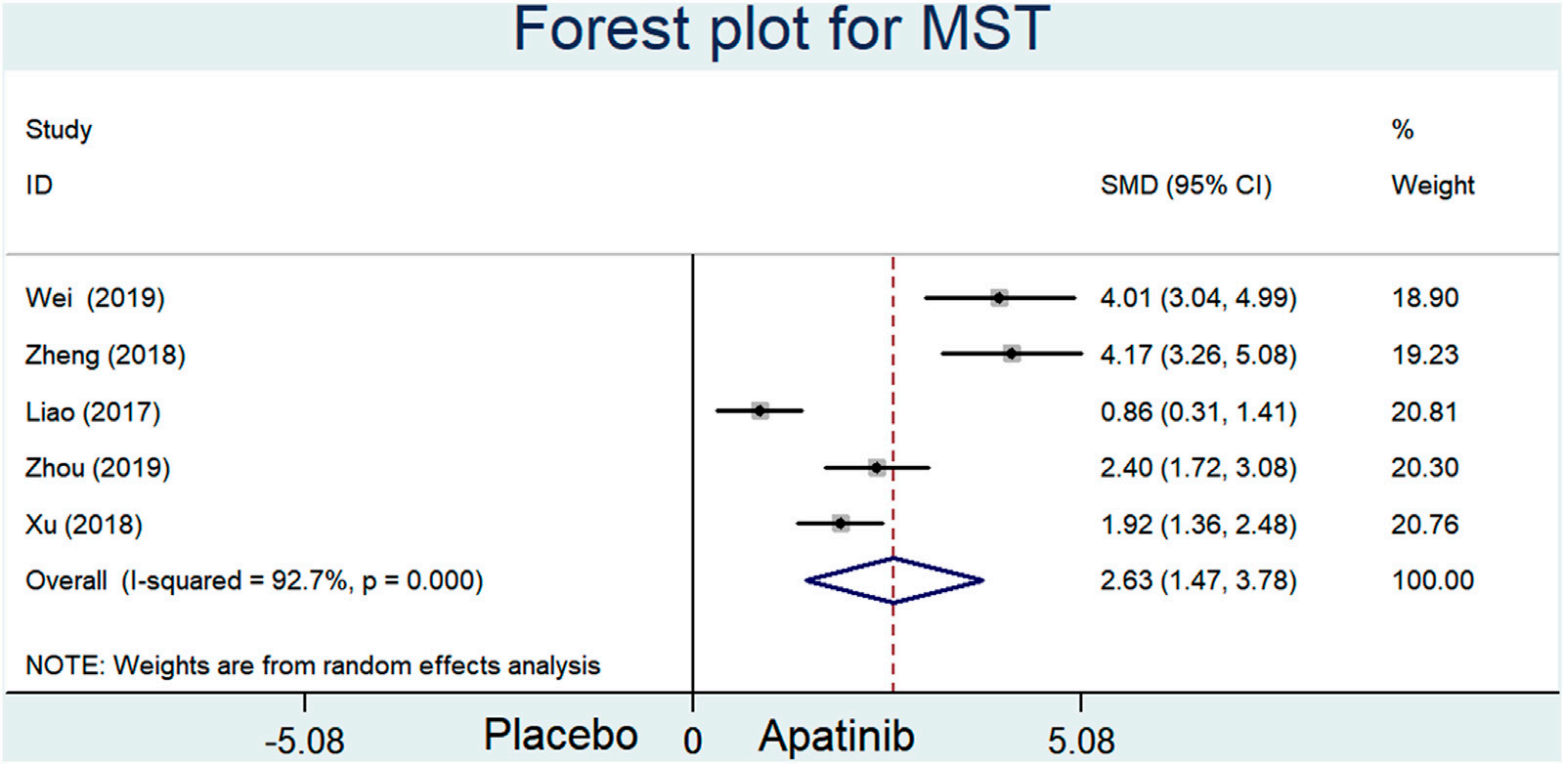
Forest plot for MST in included studies comparing apatinib with placebo.

MST data were extracted from five trials comparing apatinib with placebo. The results of our meta-analysis showed that the MST of the apatinib group was longer than that of the placebo group (SMD = 2.63; 95% CI = 1.47–3.78, *p* < 0.0001, I^2^ = 92.7%) (Figure 4).

### Meta-regression analyses for MST

Because of the significant heterogeneity in the meta-analysis for MST, univariate meta-regression analysis was applied to explore the sources of heterogeneity (Table 5). The duration of intervention (*β* = 1.696, *p* = 0.0001) evidently affected the effect size for MST. The remaining covariates, namely, year of publication (*β* = 1.03, *p* = 0.170), sample size (*β* = −0.054, *p* = 0.570), daily dose (*β* = −0.010, *p* = 0.439), and duration of follow-up (*β* = −0.218, *p* = 0.079), did not exert any influence on MST.

**TABLE 5:** Random-effect univariate meta-regression analyses of covariates on MST.

Covariate	Number of estimates	Coefficient	Lower 95% CI	Higher 95% CI	*p*
Year of publication	5	1.03	−0.442	2.50	0.170
Sample size	5	−0.054	−0.241	0.133	0.570
Daily dose (mg)	5	−0.010	−0.037	0.016	0.439
Duration of intervention (months)	3	1.696	0.743	2.648	0.0001*
Duration of follow-up (months)	3	−0.218	−0.461	0.025	0.079

CI, confidence interval; *p < 0.05.

### 6-Month and 1-year survival

**FIGURE 5:**
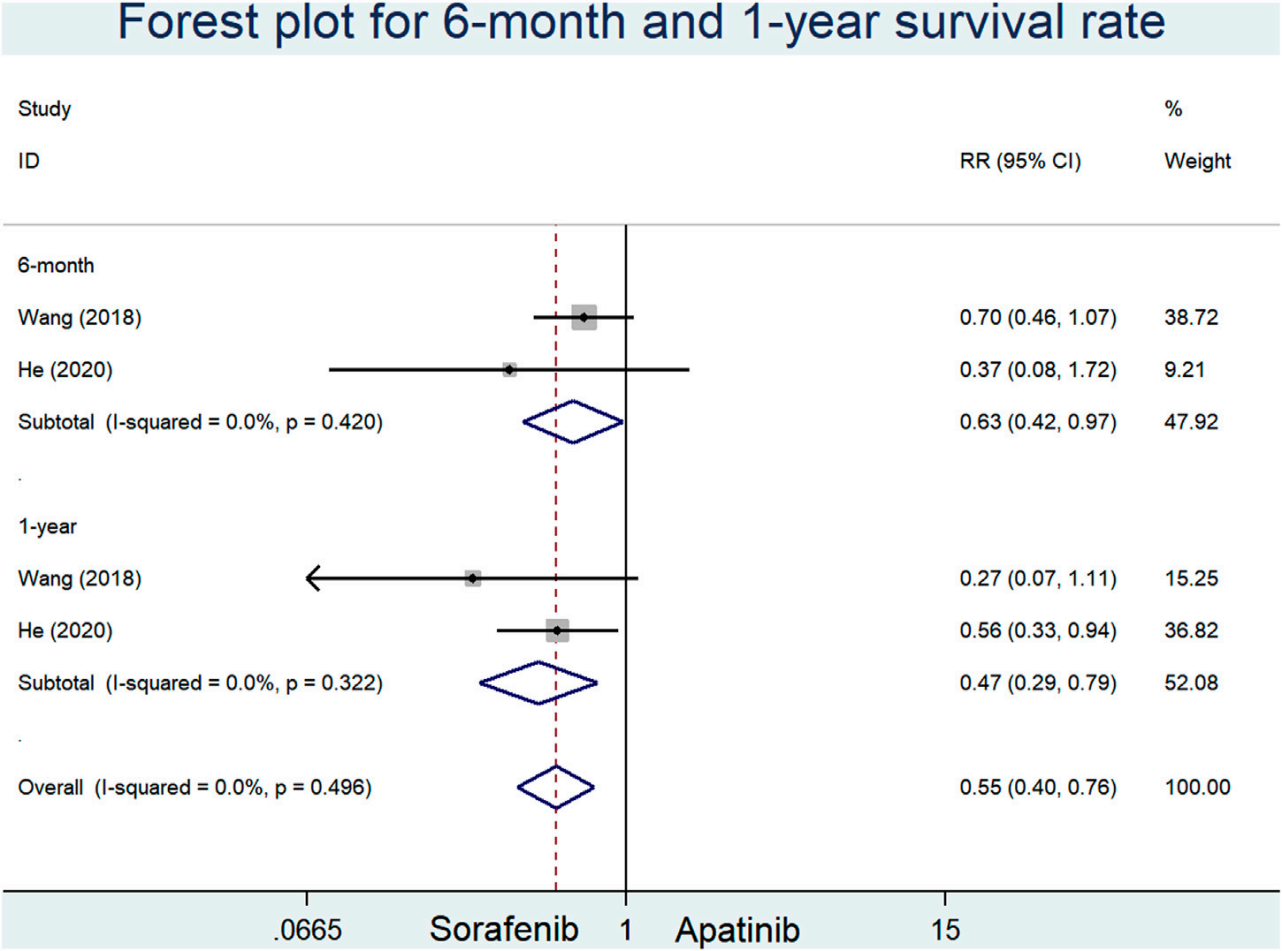
Forest plots for 6-month and 1-year survival rates in included studies comparing apatinib with sorafenib in the first-line setting.

Two RCSs reported the 6-month and 1-year survival rates of apatinib versus sorafenib in the first-line setting. Our results indicated that sorafenib significantly augmented the 6-month and 1-year survival rates in comparison with apatinib (RR = 0.63, 95% CI = 0.42–0.97, *p* = 0.036, I^2^ = 0.0%; RR = 0.47, 95% CI = 0.29–0.79, *p* < 0.0001, I^2^ = 0.0%, respectively) Figure 5

### Incidence of proteinuria

**FIGURE 6:**
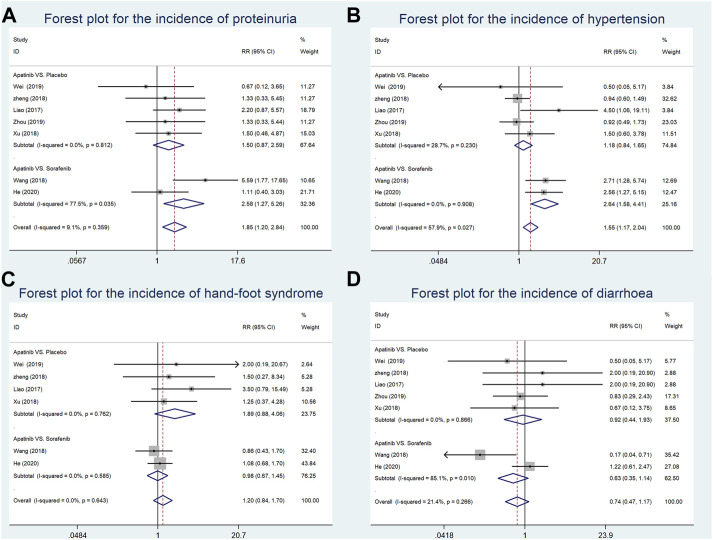
Forest plots for the incidence of adverse events in included studies in the first-line setting. **(A)** Meta-analysis for incidence of proteinuria; **(B)** meta-analysis for incidence of hypertension; **(C)** meta-analysis for incidence of hand–foot syndrome; **(D)** meta-analysis for incidence of diarrhea. RR indicates the ratio of the incidence of each outcome; 95% confidence interval is presented as error bars.

The incidence of proteinuria was reported in seven studies (four RCTs and one CCT comparing apatinib with placebo and two RCSs comparing apatinib with sorafenib). Our findings showed that the incidence of proteinuria in the apatinib group was higher than that in the sorafenib group (RR = 2.58, 95% CI = 1.27–5.26, *p* = 0.009, I^2^ = 77.5%). Moreover, there was no significant difference in the incidence of proteinuria between the apatinib and placebo groups (RR = 1.50, 95% CI = 0.87–2.59, *p* = 0.146, I^2^ = 0.0%) (Figure 6A).

### Incidence of hypertension

The data about the incidence of hypertension were extracted from seven studies (four RCTs and one CCT comparing apatinib with placebo and two RCSs comparing apatinib with sorafenib). Our results suggested that compared with sorafenib, apatinib significantly enhanced the incidence of hypertension (RR = 2.64, 95% CI = 1.58–4.41, *p* = 0.339, I^2^ = 0.0%). The incidence of proteinuria in the apatinib group was not significantly increased in comparison with the placebo group (RR = 1.18, 95% CI = 0.84–1.65, *p* < 0.0001, I^2^ = 28.7%) (Figure 6B).

### Incidence of hand–foot syndrome

The incidence of hand–foot syndrome was reported in six studies (three RCTs and one CCT comparing apatinib with placebo and two RCSs comparing apatinib with sorafenib). Apatinib showed a similar incidence of hand–foot syndrome with either placebo or sorafenib in a first-line setting (RR = 1.89, 95% CI = 0.88–4.06, *p* = 0.104, I^2^ = 0.0%; RR = 0.98, 95% CI = 0.67–1.45, *p* = 0.937, I^2^ = 0.0%, respectively) (Figure 6C).

### Incidence of diarrhea

Seven studies (four RCTs and one CCT comparing apatinib with placebo and two RCSs comparing apatinib with sorafenib) reported the incidence of diarrhea. Compared with either placebo or sorafenib, apatinib had no significant difference in the incidence of diarrhea (RR = 0.92, 95% CI = 0.44–1.93, *p* = 0.832, I^2^ = 0.0%; RR = 0.63, 95% CI = 0.35–1.14, *p* = 0.127, I^2^ = 85.1%, respectively) (Figure 6D).

### Publication bias and influence analyses

Results from Begg’s test (Z = 0.60, *p* = 0.548) and Egger’s test (t = 1.20, *p* = 0.283) showed that no significant publication biases were found among the seven studies included (Supplementary Figure S1). The funnel plot with pseudo 95% confidence limits was basically symmetric, indicating no publication bias in the results of this meta-analysis (Supplementary Figure S2). For influence analyses, we performed an additional meta-analysis for each outcome after excluding one of the original studies. We found that every single excluded trial did not evidently affect the overall results (Supplementary Figure S3), suggesting that the results of the current meta-analyses were stable and reliable.

## Discussion

This was the first comprehensive meta-analysis comparing apatinib with either placebo or sorafenib in the first-line treatment of patients with intermediate and advanced PLC. Our meta-analysis indicated that apatinib led to a higher ORR, DCR, and MST than placebo. However, compared with sorafenib, apatinib possessed a lower 6-month and 1-year survival rate and achieved a similar ORR and DCR in the first-line setting. On the other hand, apatinib had no significant upregulation of the incidence of proteinuria, hypertension, hand–foot syndrome, and diarrhea over placebo, but had a higher incidence of proteinuria and hypertension over sorafenib.

Two previous meta-analyses by Wei et al.(2019); Ye et al. (2020) reported that apatinib improved the short-term response and survival time with tolerable adverse effects in patients with intermediate and advanced HCC by comparing transarterial chemoembolization (TACE) alone with the combination therapy of TACE and apatinib. A similar result was also found in another meta-analysis including only two trials (Xue et al., 2018). These meta-analyses did not assess the benefits and harms of apatinib alone versus placebo as the first-line treatment in intermediate and advanced PLC. Furthermore, the meta-analysis did not verify whether apatinib has better therapeutic effects and safety than sorafenib in a first-line setting. The current systematic review would be a timely and important supplement in this regard.

In this meta-analysis, apatinib showed a superior advantage compared with placebo in short-term response and survival benefits. Specific oncogenic kinase targets including epidermal growth factor receptor (EGFR) and vascular endothelial growth factor receptor (VEGFR) were involved in the invasion and metastasis of malignant tumors (Hanahan and Weinberg., 2011; Kittler and Tschandl, 2018). High vascularization and over-expression of VEGF/VEGFR were discovered in patients with HCC. Increased serum VEGF was related to angiogenic activity and worse prognosis (Chao et al., 2003). Apatinib emerged as the conspicuous VEGFR2-TKI and anti-angiogenic agent for patients with PLC and could inhibit proliferation and induce apoptosis in hepatoma carcinoma cells (Zhang et al., 2018; Wen, 2019), in addition to limiting the angiogenesis of tumor tissues (He et al., 2020). Apatinib interfered with tumor growth *via* the upregulation of 3-hydroxybutyric acid (3-HB) and the increased utilization of fatty acids in the liver (Feng et al., 2019). Moreover, apatinib contributed to improving the immunosuppression of the tumor microenvironment (TME) in combination with ICIs such as anti-PD-1/PD-L1 (Zhao et al., 2019). The TME contains multiple immune suppressive cytokines such as IL-10 and TGF-β that mediate T-cell dysfunction (Mukaida and Nakamoto., 2018). Immunosuppression could develop acquired resistance in patients with ICI monotherapy.

For a decade, clinical studies with ICI therapies were widely performed with important breakthrough. Immunotherapy based on ICIs provided a great prospective approach in the treatment of HCC. ICIs could activate T cells and NK cells from priming by antigen-presenting cells (APCs) *via* mediating the ligand–receptor pairs such as CTLA-4-CD80/86 and PD-1-PD-L1/PD-L2 in order to induce tumor regression (Dyck and Mils., 2017) (Leone et al., 2021). Although apatinib was not a better option than sorafenib in this meta-analysis, many studies showed that apatinib as the synergistic agent of ICIs could bring potential survival benefits in HCC. Apatinib combined with camrelizumab (anti-PD-1 antibody) showed significant efficacy in patients with advanced HCC, and the 12-month survival rate reached 74.7% (95% CI = 62.5–83.5) (Xu et al., 2021). Apatinib plus capecitabine combined with sintilimab (anti-PD-1 antibody) provided great therapeutic effects and safety for unresectable HCC in the first-line treatment (Li et al., 2022). Nevertheless, sorafenib could damage the activity of ICIs *via* inhibiting major histocompatibility complex class I in cancer cells, so that was not suitable for use in combination with ICIs (Zhao et al., 2016) (Hage et al., 2019). Meanwhile, the combination of ICIs with other treatments, including TKIs, inhibitors of angiogenesis, loco-regional therapies, or chemotherapies, had also shown promising therapeutic action in HCC, with low cytotoxicity and lasting response (Finn et al., 2020) (Duffy et al., 2017) (Lee et al., 2020) (Qin et al., 2019).

In this meta-analysis, there was no significant difference between apatinib and placebo in the incidence rate of non-hematologic toxicities including proteinuria, hypertension, hand–foot syndrome, diarrhea, and asthenia and hematologic toxicities such as neutropenia, thrombocytopenia, leukopenia, and hemorrhage (Supplementary Figure S4), whereas apatinib showed a greater incidence in non-hematologic toxicities including hypertension and proteinuria compared with sorafenib in the first-line setting. Unfortunately, this meta-analysis could not analyze the difference of incidence in hematologic toxicities between apatinib and sorafenib due to inadequate data.

In order to avoid the withdrawal or interruption of treatment, apatinib-related adverse events still required attention. Several previous trials suggested that apatinib-induced toxic effects were manageable through dose interruptions or reductions (Hu et al., 2014; Song et al., 2017). If necessary, symptomatic treatment could serve as an important clinical option. Proteinuria was a vital indicator of renal injury and the most frequent renal side effect induced by anti-VEGF drugs. Anti-VEGFR drugs could promote renal thrombotic microangiopathy and synergistically affect the capacity of glomerular filtration with consequent proteinuria (Izzedine et al., 2007; Eremina et al., 2008). Hypertension might be involved in the decreased secretion of vasodilators and incremental vascular resistance (Facemire et al., 2009). Hand–foot syndrome could correlate with the repression of VEGFR, which exerted a negative impact on endothelial cells and led to damage of localized tissues (Miller et al., 2014; McLellan et al., 2015). In addition, severe diarrhea would cause electrolyte disorders and dehydration. In this case, besides oral antidiarrheal agents, intravenous fluids were also needed to replenish amino acid, protein, ion, and water in patients. Clinical treatment normally prevented and reduced hematological toxicities *via* stimulating bone marrow hematopoietic stem cells and their microcirculation.

There were other possible limitations to this meta-analysis. First, diagnostic criteria and study design were inconsistent in the original studies. Second, the information about characteristics of subjects included such as duration of treatment, follow-up period, and tumor size was incomplete, and the quality assessment score was low in some studies. Lastly, these subjects included were limited to the Asian population, so international, large, and multi-center studies were necessary to confirm our findings.

## Conclusion

In general, this systematic review and meta-analysis indicated that apatinib may be an additional treatment option for patients with intermediate and advanced PLC in the first-line setting. Sorafenib alone showed a better survival rate within 1-year and a lower incidence rate in hypertension and proteinuria than apatinib monotherapy.
