# Supplementary material for: Efficacy and safety of apatinib versus sorafenib/placebo in first-line treatment for intermediate and advanced primary liver cancer: A systematic review and meta-analysis
Source: Front Pharmacol. 2023 Apr 21;14:1101063. doi: 10.3389/fphar.2023.1101063 (PMC10160361; doi:10.3389/fphar.2023.1101063)

Supplementary Material

## Supplementary Figures

**Supplementary Figure S1:** A graph from Egger’s test.


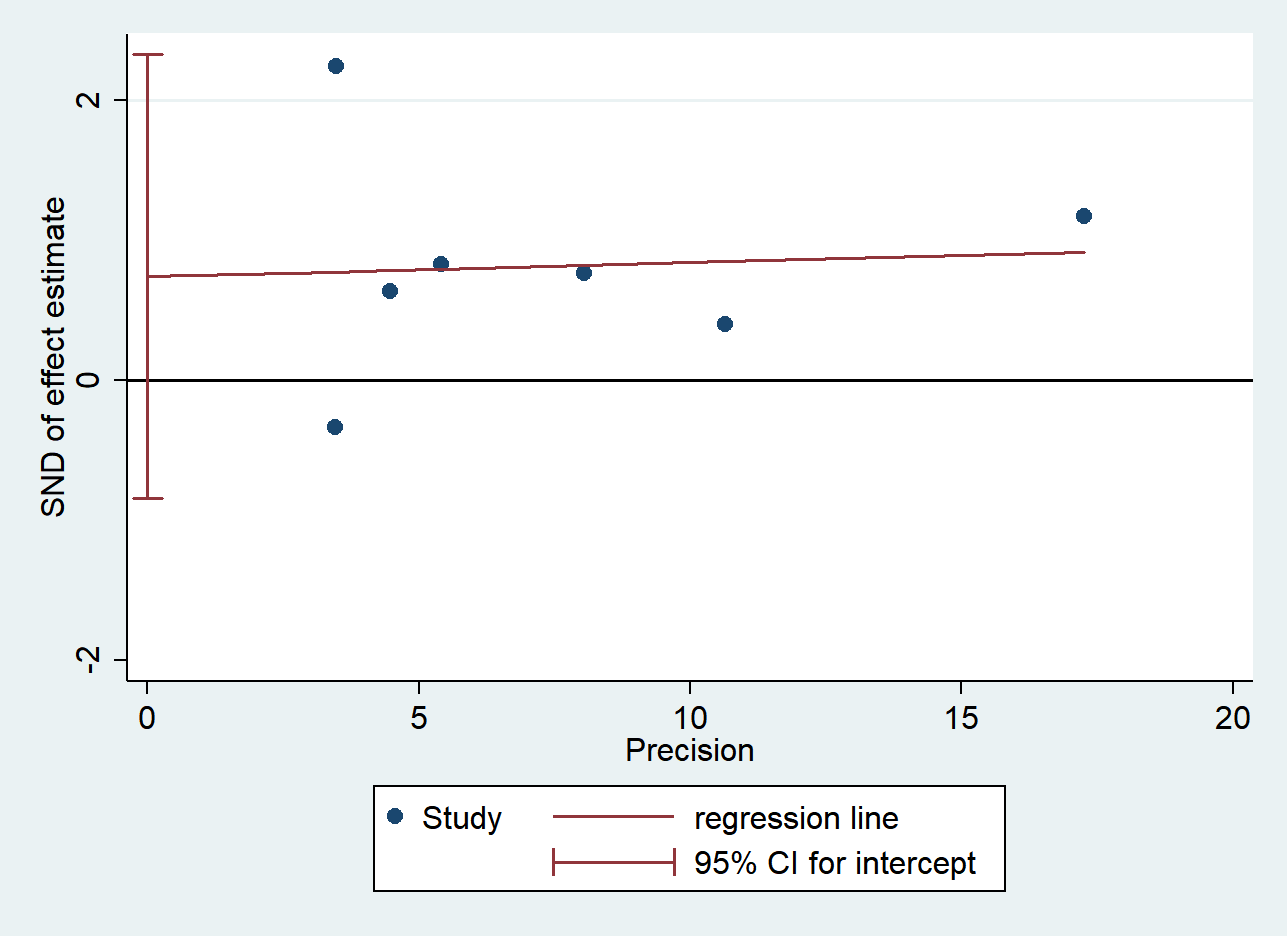


**Supplementary Figure S2:** Funnel plot with pseudo 95% confidence limits.


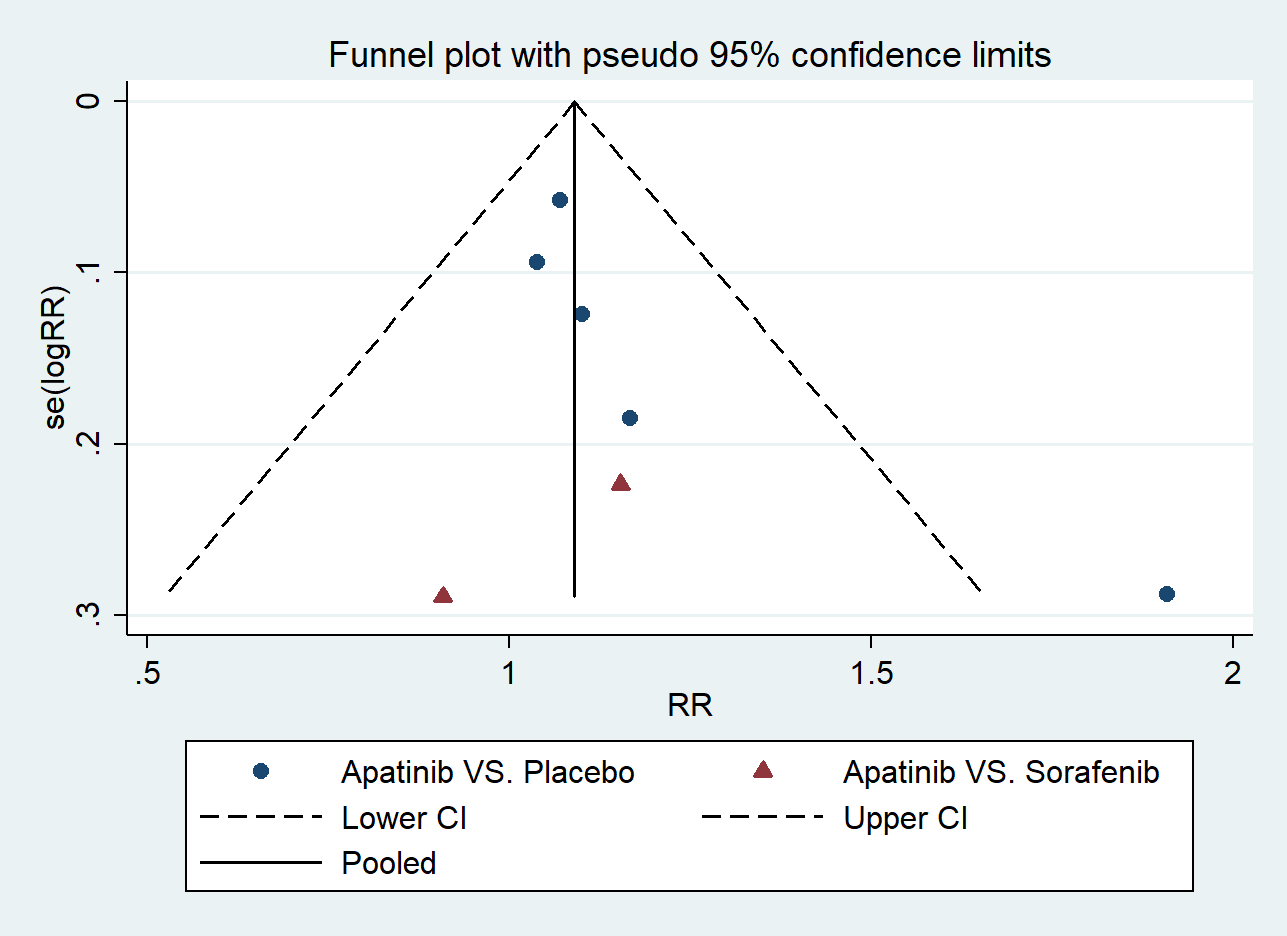


**Supplementary Figure S3:** Sensitivity analyses about 5 studies comparing apatinib with placebo.


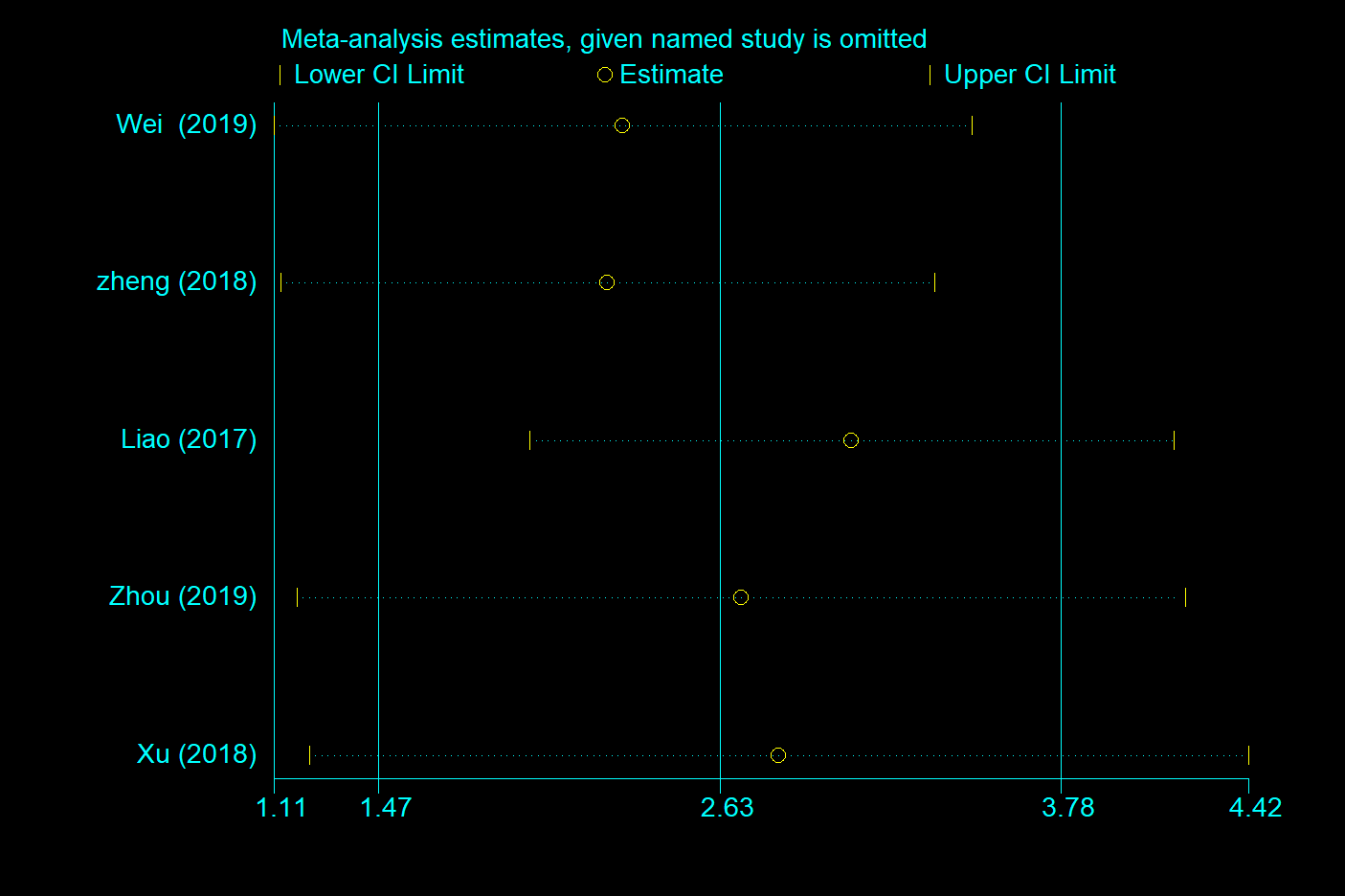


**Supplementary Figure S4.** Forest plots for the incidence rate of partial adverse events in 5 studies comparing apatinib with placebo.


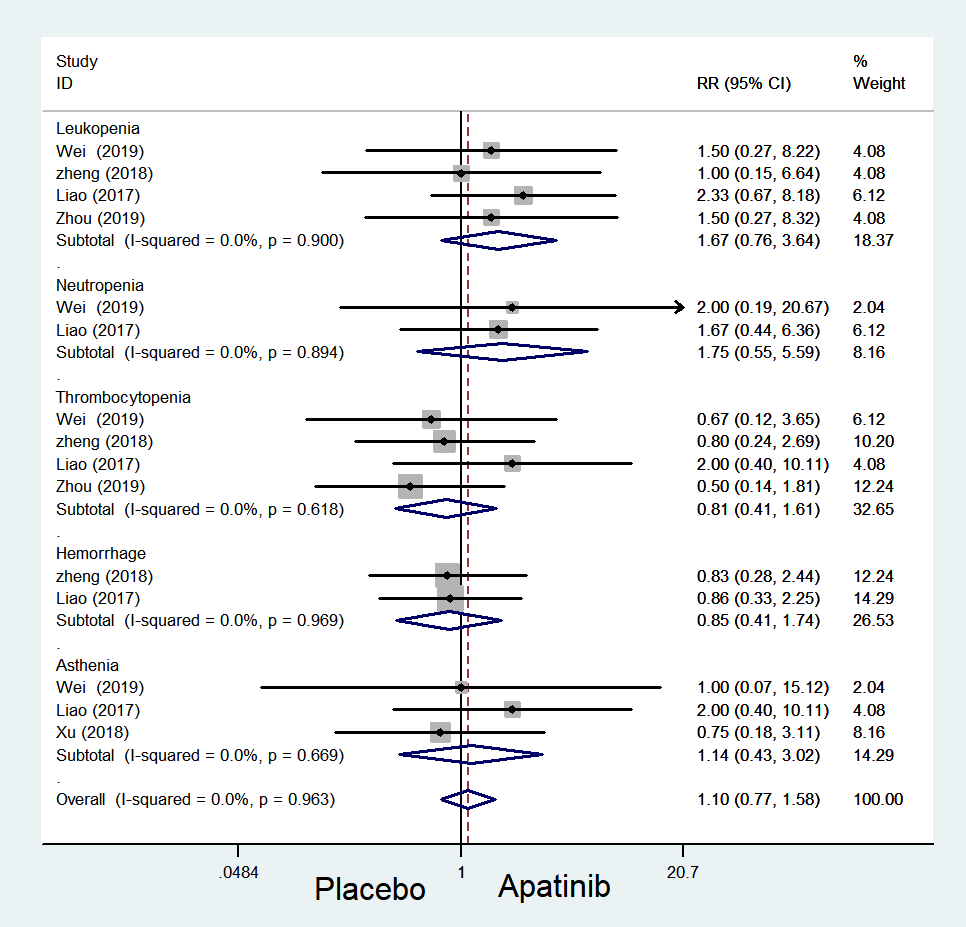

Supplement: Supplementary file 2 [file DataSheet1.DOCX]
